# A Long-Term Safety and Efficacy Report on Intravitreal Delivery of Adipose Stem Cells and Secretome on Visual Deficits After Traumatic Brain Injury

**DOI:** 10.1167/tvst.11.10.1

**Published:** 2022-10-03

**Authors:** Pratheepa Kumari Rasiah, Kumar Abhiram Jha, Jordy Gentry, Nobel A. Del Mar, Tanisha Townsend, Kwame E. Torgbe, Anton Reiner, Rajashekhar Gangaraju

**Affiliations:** 1 Department of Ophthalmology, University of Tennessee Health Science Center, Memphis, TN, USA; 2 Department of Anatomy & Neurobiology, University of Tennessee Health Science Center, Memphis, TN, USA; 3 Department of Pathology, University of Tennessee Health Science Center, Memphis, TN, USA

**Keywords:** mesenchymal stem cells, blast injury, retina, GFAP, inflammation

## Abstract

**Purpose:**

We compared intravitreal injection of human adipose stem cell concentrated conditioned media (ASC-CCM) to injection of live ASCs for their long-term safety and effectiveness against the visual deficits of mild traumatic brain injury (mTBI).

**Methods:**

We first tested different intravitreal ASC doses for safety. Other C57BL/6 mice then received focal cranial blast mTBI and were injected with the safe ASC dose (1000 cells/eye), ASC-CCM (∼200 ng protein/eye), or saline solution. At five and 10 months after blast injury, visual, molecular, and histological assessments evaluated treatment efficacy. Histological evaluation of eyes and other organs at 10 months after blast injury assessed safety.

**Results:**

Human ASCs at 1000 cells/eye were found to be safe, with >10,000 cells causing retinal damage. Blast-injured mice showed significant vision deficits compared to sham blast mice up to 10 months. Blast mice receiving ASC or ASC-CCM showed improved vision at five months but marginal effects at 10 months, correlated with changes in glial fibrillary acidic protein and proinflammatory gene expression in retina. Immunostaining for human IgG failed to detect ASCs in retina. Peripheral organs examined histologically at 10 months after blast injury were normal.

**Conclusions:**

Intravitreal injection of ASCs or ASC-CCM is safe and effective against the visual deficits of mTBI. Considering the unimproved glial response and the risk of retinal damage with live cells, our studies suggest that ASC-CCM has better safety and effectiveness than live cells for the treatment of visual dysfunction in mTBI.

**Translational Relevance:**

This study demonstrates the safety and efficacy of mesenchymal stem cell-based therapeutics, supporting them for phase 1 clinical studies.

## Introduction

Mesenchymal stem cells (MSCs) and their derivatives have been recently explored as treatments in a variety of ocular diseases, with few therapies having shown safety and efficacy in clinical trials following promising preclinical studies.^1^ Among the various sources of MSCs, we and others have shown significant progress with adipose tissue (adipose stem cell [ASC]), or bone marrow–derived MSCs, as a potential therapeutic in a variety of eye diseases,^2^^-^^4^ including traumatic brain injury (TBI).^5^^-^^7^ Stem cell transplantation strategies for retinal diseases could be broadly categorized into the following: (1) direct delivery of stem cell-derived progenitors or molding them into implantable sheets such that the stem cells integrate with the impaired retina mainly by reconstruction of the inner retina circuitry^8^ or enhanced integration of transplanted cells for tissue remodeling^9^; and (2) direct delivery of stem cells for their secreted factors (secretome) to combat the inflammatory^10^^,^^11^ or oxidative stress-causing milieu.^12^ Although MSCs directly delivered into the vitreous space in some animal models have shown successful integration into the host tissue, the retention rates were poor, in some cases the benefits were noted even in the absence of integration of such transplanted MSCs.^11^^,^^13^^,^^14^ More importantly, several recent clinical studies using MSCs have shown a high rate of complications associated with delivery procedures,^15^ including retinal detachment,^16^^,^^17^ and complete blindness.^18^ These clinical observations were also reproduced in some preclinical experiments,^19^^,^^20^ suggesting that direct implantation of MSC therapeutics has limited compatibility with the vitreous domain of the eye.

Stem cell therapies without proper scientific validation, proof of efficiency, and safety create an undue burden on regulatory bodies to approve such research innovations and protect the public from harm.^21^ Because MSCs are an excellent source for beneficial paracrine factors, several studies have explored the delivery of the stem cell secretome as an alternative to injecting live stem cells.^22^^,^^23^ Although secretome-based therapies have shown tremendous promise, their long-term safety and efficacy have not been explored, specifically in TBI models. Our previous studies have provided evidence for a short-term efficacy of the ASC secretome (ASC-concentrated conditioned medium [ASC-CCM]) in protecting against the visual dysfunction and retinal neurodegeneration after a closed head mild TBI (Mild Traumatic Brain Injury (mTBI)) and direct ocular blast injury models.^7^^,^^10^^,^^12^^,^^24^ The primary objective of the current study is to assess the long-term effects of secretome therapy in a mouse model of mTBI. The secondary objective of the study is to determine the safe limits of live stem cell transplantation (ASCs) in a mouse model of mTBI.

## Methods

### Animal Care

All animal procedures were approved by the Institutional Animal Care and Use Committee, UTHSC, Memphis, and USAMRMC Animal Care and Use Review Office, and followed the guidelines of the Association for Research in Vision and Ophthalmology Statement for the Use of Animals in Ophthalmic and Vision Research. Twelve-week-old C57BL/6J mice were purchased from The Jackson Laboratory (Bar Harbor, ME, USA) and maintained in a controlled temperature environment with a 12-hour dark/12-hour light cycle, and with access to food and water as desired.

### Adipose Stem Cell Culture and ASC-CCM Preparation

Adipose tissue–derived stem cells were purchased from Lonza (Walkersville, MD, USA). Cells were cultured in EGM2-MV media in T75 flasks at a density of 5.3 × 10^3^ cells/cm^2^ at 37°C and 5% CO_2_. Cells were trypsinized, briefly washed with saline, and cell viability was determined. The number of ASCs was adjusted to deliver 1000 cells in 2 µL volume/eye for intravitreal injections for blast TBI mice. ASC-CCM was prepared from ASCs as described earlier.^10^ Briefly, the cells were washed twice with Dulbecco's Phosphate Buffered Saline (DPBS) and stimulated with 20 ng/mL TNFα (R&D Systems, Minneapolis, MN, USA) and 10 ng/mL IFNγ (R&D Systems) in basal MEM media (Thermofisher, St. Louis, MO, USA). After 24 hours, the exogenous cytokines were removed by washing twice with DPBS. The culture media was replaced with basal MEM media. After a further 24 hours, cell-free supernatant was collected, concentrated with a 3-kDa molecular weight cutoff Amicon Ultra-15 centrifugal concentrator (Millipore Sigma, Burlington, MA, USA), and desalted against DPBS. The protein concentration was determined using Qubit Protein Assay Kit (Thermofisher).

### Dose Comparison Study of ASC

**Figure 1.**
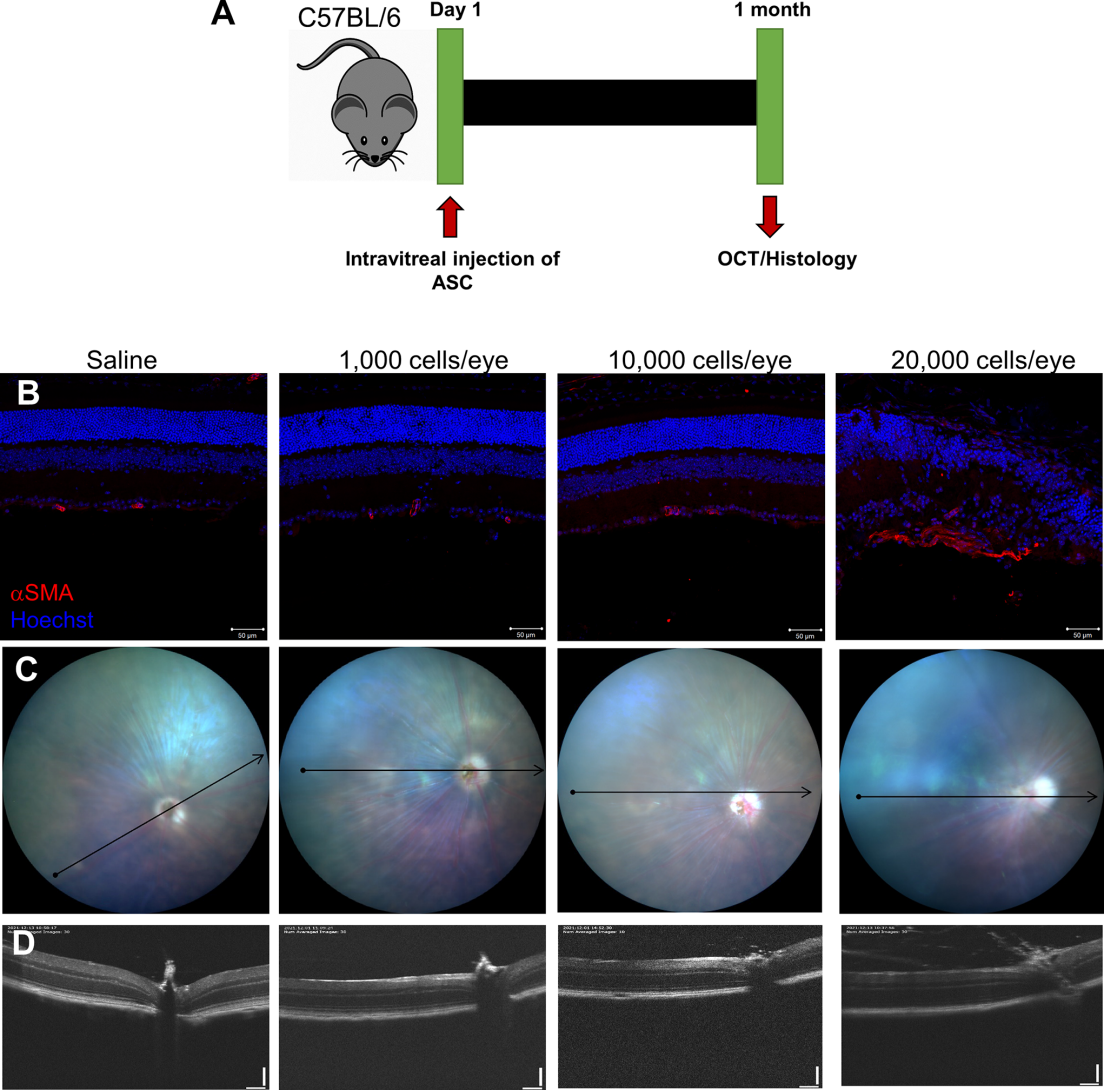
Intravitreal injection of ASC at a low dose is safe, whereas higher doses cause retinal damage. **(A)**. Timeline of ASC delivery and follow-up assays. **(B)**. Representative confocal immunofluorescence images of C57BL/6 mice retina intravitreally injected with saline or increasing dose of ASCs. *Scale bar:* 50 µm. **(C)**. Representative en-face images of OCT scan showing the b-scan orientation (*arrow*). **(D)**. Representative b-scan images from different groups. Data shown from one animal representing n = 5 animals/group.

To determine the safe dose of ASCs, C57BL/6 mice were injected with one of the following concentrations 0; 1000; 10,000 or 20,000 cells/eye in 2 µL volume/eye and monitored their integration, and the structural integrity of the retina by immunohistochemistry and optical coherence tomography (OCT) (Phoenix-Micron, Inc, Bend, OR, USA), respectively. For cell tracking purposes, ASCs were labeled with Vybrant DiO Cell-Labeling Solution (Thermofisher) for 30 minutes in serum-free media, washed with phosphate-buffered saline solution (PBS), and suspended in an appropriate volume of saline solution (maximum injection volume 2 µL) for intravitreal injections. After four weeks, animals were anesthetized to perform OCT as described below, followed by euthanasia to collect retina for tissue processing, immunohistology, and confocal imaging (Fig. 1).

### mTBI Experiment

Animals were randomized into four groups: (1) sham-blast-saline, (2) blast-saline, (3) blast-ASC, and (4) blast-ASC-CCM. About 24 hours before the blast injury, animals were provided with 32 mg/mL acetaminophen suspension (Infant Tylenol, cherry flavor; Johnson & Johnson, New Brunswick, NJ, USA) provided in drinking water, yielding a dose of 300 mg/kg/d and continued until 48 hours after the blast injury. Mild TBI was performed as previously described.^10^^,^^25^ Briefly, mice were anesthetized with a subcutaneous dose of ketamine (50 mg/kg; Henry Schein Medical Supplies, Novi, MI, USA) and dexmedetomidine (0.25 mg/kg; Zoetis, Parsippany-Troy Hills, NJ, USA) cocktail. A single blast of 50 psi was given to each mouse in the blast groups on the left cranium between the ear and the eye. Sham-blast animals received a 0-psi blast. Within two to three hours after blast injury, eyes were dilated with 1% tropicamide and 0.5% proparacaine to inject 2 µL of sterile saline into both sham-blast and blast-saline mice. The blast-ASC mice received a bilateral intravitreal injection of 1000 cells (suspended in sterile saline). On the other hand, the Blast-ASC-CCM mice received a bilateral intravitreal injection of ∼200 ng of protein, equating to 2 µL of volume. After the procedure, animal anesthesia was reversed with Atipamezole Hydrochloride (0.5 mg/kg; Zoetis).

### Optokinetic Measurements (OKN)

**Figure 2.**
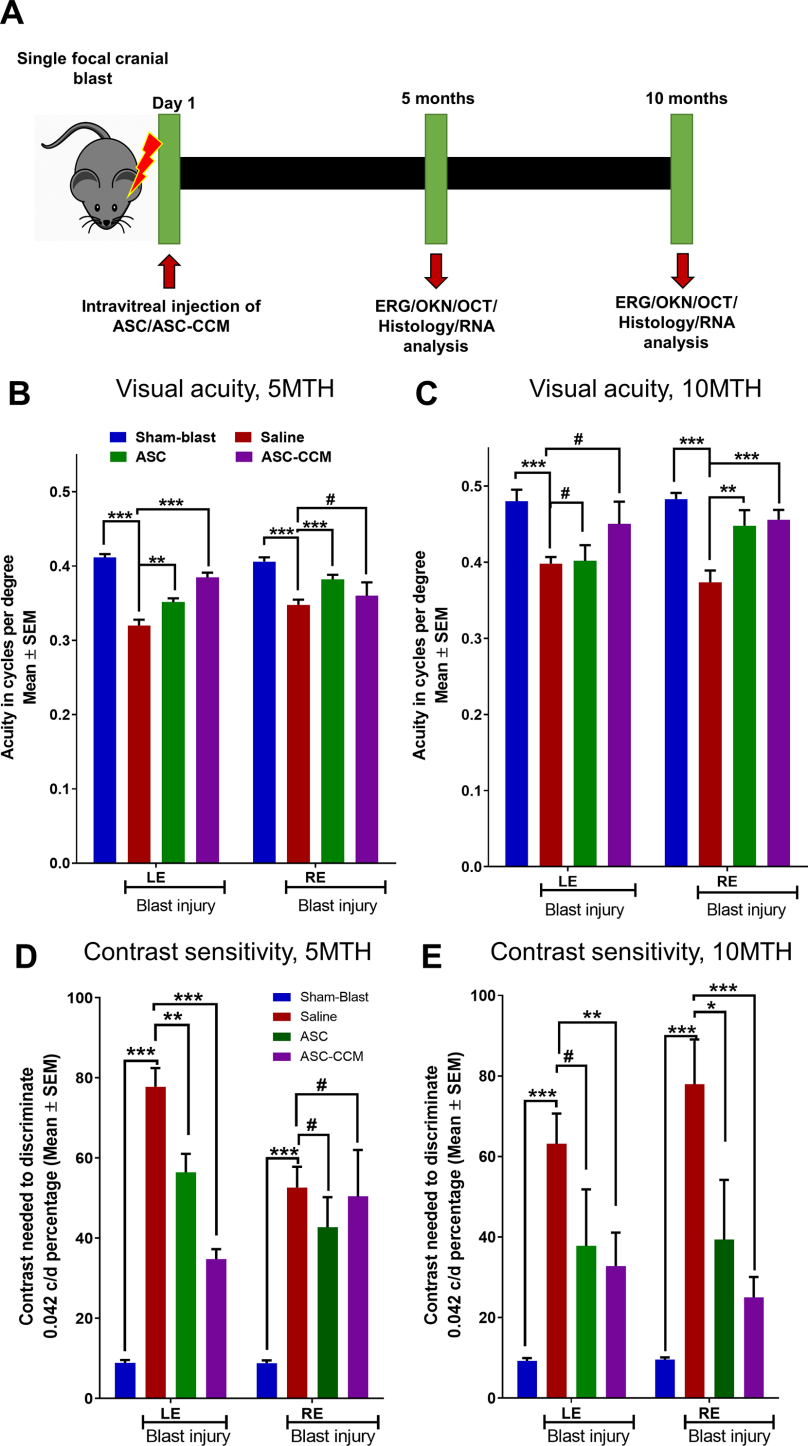
ASC-CCM and ASCs in mice subjected to blast injury improve visual acuity and contrast sensitivity thresholds. **(A)** Timeline of mTBI mouse studies, intravitreal injection of saline solution, ASC, or ASC-CCM, and endpoint analyses. **(B)** Visual acuity at five months. **(C)**. Visual acuity at 10 months, **(D)** Contrast sensitivity thresholds at five months. **(E)** Contrast sensitivity thresholds at 10 months in all groups of mice. Data represent combined mean ± SEM from n = 7–8 animals/group. *P < 0.05; **P < 0.01; ***P < 0.001; ^#^*P* > 0.05.

The visual acuity (VA) and contrast sensitivity thresholds (CS) were assessed for both eyes as previously described.^7^^,^^26^ Briefly, after the intended experimental duration (Fig. 2, the optokinetic reflex to a continuously moving field was analyzed using an OptoMotry unit (Cerebral Mechanics Inc, New York, NY, USA). Mice were placed unrestrained on the platform in the center of the experimental box and allowed to track the vertical sine wave lines on a rotating cylinder presented via an LCD display. An investigator blinded to the experimental group identified the reflexive head and neck movements (in clockwise or anticlockwise directions) of the animals in response to the moving stripes. Acuity testing was performed at 100% contrast (i.e., white versus black stripes) with varying spatial frequency of the stripes, while contrast sensitivity testing was performed at a fixed spatial frequency threshold (0.042 c/d) but varying stripe contrast.

### Electroretinography Stimulations and Recordings

**Figure 3.**
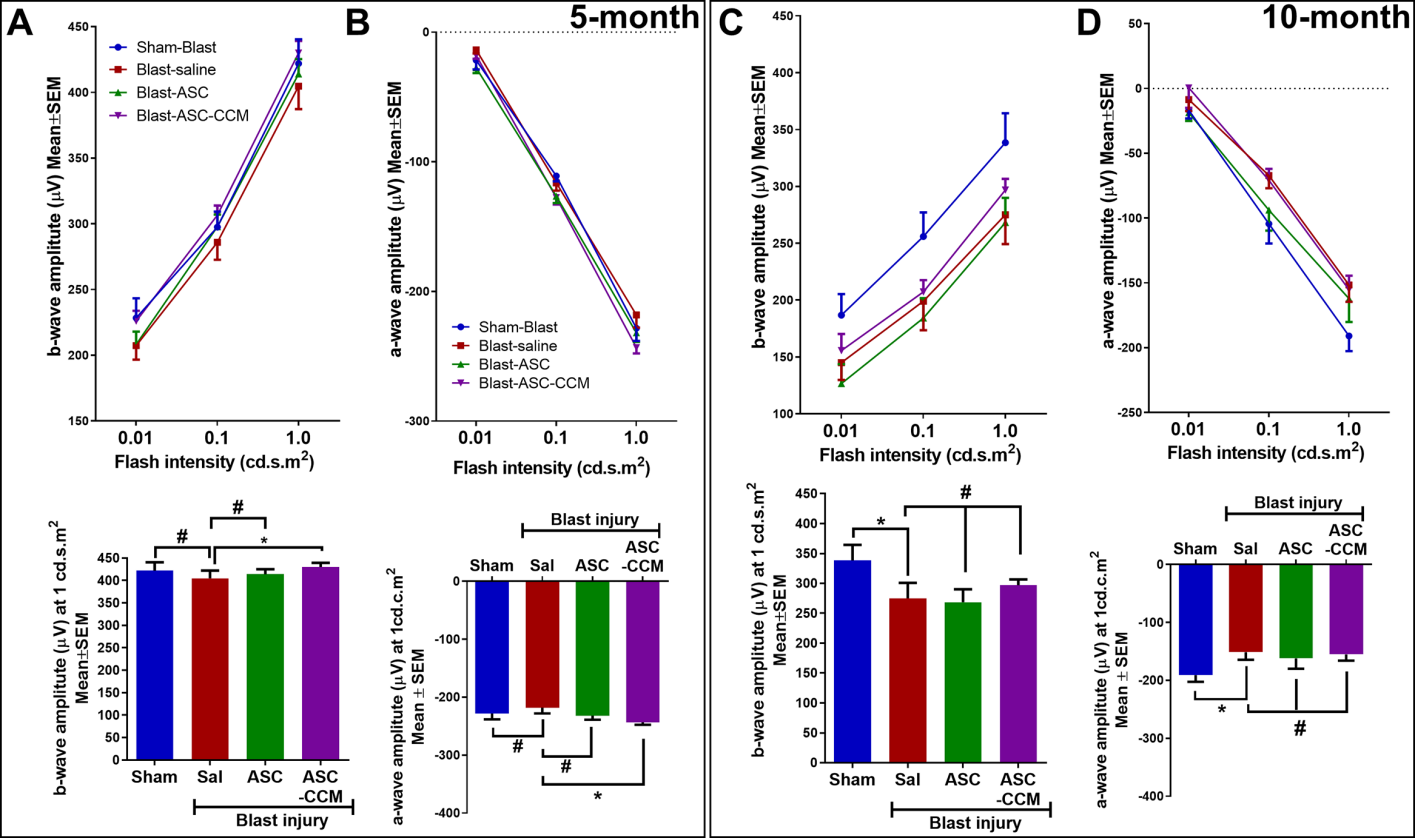
ASC-CCM and ASCs partially improve b-wave and a-wave amplitudes in mTBI mice. **(A)** Left eye b-wave amplitude measurement in mice at various flash intensities at five months. Below: b-wave amplitudes shown at 1.0 cd · s · m^2^ from all groups. **(B)** Left eye a-wave amplitudes at five months. Below: a-wave amplitudes shown at 1.0 cd · s · m^2^ from all groups. **(C)** Left eye b-wave amplitude measurement in mice at various flash intensities at 10 months. Below: b-wave amplitudes shown at 1.0 cd · s · m^2^ from all groups. **(D)** Left eye a-wave amplitudes at 10 months. Below: a-wave amplitudes shown at 1.0 cd · s · m^2^ from all groups. Data represent combined mean ± SEM from n = 10−18 animals/group (five months), n = 6−10 animals/group (10 months). *P < 0.05; ^#^*P* > 0.05.

Electroretinograms (ERGs) were recorded from both eyes as described in our previous publication.^7^ Briefly, after 5 and 10 months (Fig. 3), experimental animals were dark-adapted overnight and anesthetized with a ketamine (50 mg/kg) and dexmedetomidine (0.25 mg/kg) cocktail. Pupil dilation was achieved with 1% tropicamide (Bausch & Lomb). The dual light guide electrodes with stimulator were positioned on the surface of both corneas. Light pulses were delivered at 0.01, 0.1, and 1.0 cd-s/m^2^, and the responses were recorded simultaneously from both eyes (Celeris Rodent Electrophysiology; Diagnosys LLC, Lowell, MA, USA). All offline analyses were conducted with Diagnosys software. A minimum of five responses to a given light stimulus intensity were averaged to determine the a- and b-wave amplitudes.

### OCT

OCT was performed for both eyes using a Micron IV Image-Guided OCT system for rodents (Phoenix-Micron, Inc). Mice that had been deeply anesthetized using ketamine (50 mg/kg; Henry Schein Medical Supplies) and dexmedetomidine (0.25 mg/kg; Zoetis) cocktail were placed on the platform that allowed movements in X, Y, and Z planes. Eyes were dilated with 1% Tropicamide, and retinal layers were imaged with the Reveal Micron OCT software.

### Tissue Processing, Immunohistology, and Confocal Imaging

After physiological data were collected, some animals used to evaluate ASC and ASC-CCM efficacy after blast TBI were euthanized with inhalation of CO_2_; and whole eye globes were enucleated and fixed with warm 4% paraformaldehyde in 0.1 phosphate buffer (PB) overnight at 4°C. After this, the tissues were cryoprotected in 10% to 30% sucrose in 0.1 PBS and embedded in optimal cutting temperature embedding media (Tissue-Tek; Sakura Finetek USA, Inc., Torrance, CA, USA). Serial sections of 12 µm thickness were collected on Superfrost/Plus microscope slides.

The sections were air-dried and washed with 1 × PBS briefly, followed by blocking with normal goat serum and bovine serum albumin in 0.3% TritonX100 solution. Primary antibody incubation was carried out overnight (1:1000 Anti-Glial Fibrillary Acidic Protein, Z0334; Dako, Glostrup, Denmark), (1:1000 anti-Human IgG, ab200699; Abcam, Cambridge, MA, USA), (1:500 anti-alpha-smooth muscle actin [αSMA], ab5694; Abcam) in the blocking buffer. The primary antibody was detected with appropriate Alexa Fluor conjugated secondary antibodies (Thermofisher; at a 1: 1000 dilution). Sections were counter-stained with Hoechst/DAPI to identify cells by their nuclei. Imaging was done with Zeiss LSM 710 laser scanning confocal microscope with a ×20 objective and 1.0 zoom. Z- stack images were captured to encompass the thickness of the tissue section; a maximum intensity projection was processed to compress the z-stacks into a single plane image. For studies involving glial fibrillary acidic protein (GFAP) immunolabeling, the pixel intensities were quantified with ImageJ software and expressed as mean intensity per 100,000 µm^2^ of the retina.

### Hematoxylin and Eosin (H&E) Staining

At the end of the experimental time points, some animals were anesthetized with Isoflurane, and transcardially perfusion fixed with 4% paraformaldehyde. Visceral organs, including brain, spleen, lung, kidney, heart, and liver, were excised and washed with PBS. Following this, tissues were fixed overnight with 10 % buffered formaldehyde (pH 7.4) at room temperature. Tissues were processed using Sakura VIP E300 automated tissue processor (Sakura Finetek USA, Inc) at the Research Histology Core Laboratory, UTHSC. Paraffin blocks were made using Thermo Histocentre 3 embedding station (Thermo Fisher Scientific, Waltham, MA, USA). Serial sections of 5 µm thickness were cut (Leica Biosystems RM2235, Leica, Germany) and were used for H&E staining (Tissue-Tek DRS 2000 Slide Stainer; Sakura Finetek USA, Inc). The stained sections were observed under a light microscope by a board-certified pathologist not involved in the study design to check for any pathological changes. Following pathological grading, images of the entire tissue sections were captured using Lionheart FX Automated Microscope (BioTek, Winooski, VT, USA) with a ×10 objective.

### RT qPCR Analysis

Retinal tissue was subjected to total RNA isolation using the NucleoSpin RNA Plus kit (Macherey-Nagel GmbH, Düren, Germany) as per the manufacturers' protocol. A two-step RT-PCR was conducted using High-Capacity cDNA Reverse Transcription Kit (Applied Biosystems), and the second step of cDNA amplification qPCR was done using Taqman probes (Table). The mRNA expression was normalized to the expression of 18S ribosomal RNA as the internal control as described by us previously.^24^

**Table.** TaqMan Assay Primer and Probes for Gene Transcript Analysis

Genes	Taqman Assay ID	Reference Sequence
18S ribosomal RNA (18s)	Mm04277571	NR_003278
Interleukin 1 beta (1L1β)	Mm00434228_m1	NM_008361.3
Cluster of Differentiation 86 (Cd86)	Mm00444543_m1	NM_019388.3
Glutamine synthetase (gs)	Mm00725701_s1	NM_008131.4
Interferon regulatory factor 8	Mm00492567_m1	AK018533.1
v-erb-b2 erythroblastic leukemia viral oncogene homolog 3 (Erbb3)	Mm01159999_m1	NM_010153.1
Fas/TNF receptor superfamily member 6 (Fas)	Mm01204974_m1	NM_007987.2
Vascular cell adhesion molecule 1 (Vcam1)	Mm01320973_m1	NM_011693
Endothelin 2 (Edn2)	Mm00432983_m1	NM_007902.2

### Statistical Analysis

Data are presented as mean ± standard error of the mean (SEM) of each group and compared to other groups*.* Data were analyzed using one-way analysis of variance followed by pairwise *t*-tests to calculate the *P* values for comparisons between the individual groups after Bonferroni correction. Plots were generated using GraphPad (Prism, La Jolla, CA, USA). In all cases, *P* < 0.05 was considered statistically significant.

## Results

### OCT and Immunohistology After Intravitreal Injection of ASC

To assess the best-tolerated dose of ASCs in a mouse eye, intravitreal injections were performed in C57BL/6 mice with either 0, 1000, 10,000, or 20,000 cells per eye under anesthesia and followed up at four weeks with OCT and immunohistology (Fig. 1A). Both fundus imaging and b-scan OCT of mice receiving intravitreal injections of 1000 and 10,000 cells (Figs. 1C, 1D) did not show any gross changes as compared to saline solution–injected mice. Both retinal thickness and gross appearance of the vasculature were indistinguishable among 1000 cell–, 10,000 cell–, and saline solution–injected groups, with the exception of one animal in the 10,000 cells group. Specifically, one of the five mice that received 10,000 cells demonstrated a cluster of DiO-labeled ASCs closer to the optic cup and an engorged vasculature as evidenced by OCT (Supplementary Fig. S1). Retinal sections stained with DAPI and immunostained with αSMA demonstrated a near normal thickness of the retina and very few αSMA-positive capillaries in the ganglion cell layer (GCL), thus confirming the OCT observations (Fig. 1B). Interestingly, DiO-labeled transplanted cells could not be detected in any mice in either the vitreous or throughout the retina except for the one animal in 10,000 cells group, which was double positive for αSMA and DiO (Supplementary Fig. S1). In contrast, five out of five mice that received 20,000 cells as noted by fundus imaging and OCT demonstrated several abnormalities including engorged vasculature, vitreous or optic cup deposition of cells, and retinal outgrowth (Figs. 1C, 1D). Moreover, in this group of animals, retinal sections stained with DAPI and immunostained with αSMA demonstrated retinal thinning, retinal invagination, and detachment, in addition to increased αSMA-positive capillaries in GCL that were also positive for DiO labeling. Our results thus indicate that intravitreal injection of ASC at a dose of 1000 cells per retina is safe, while higher doses cause increasingly greater retinal damage (Fig. 1B, Supplementary Fig. S1). Considering that 1000 cells were well tolerated in the mice, subsequent experiments were performed by injecting 1000 cells/2 µL per eye.

### Visual Function After Intravitreal Injection of ASC-CCM and ASCs in Mice Subjected to Blast Injury

Using the focal cranial blast model of mTBI, we have previously shown that intravitreal injection of the ASC-derived secretome (ASC-CCM) suppressed retinal inflammation, microglial polarization, and Müller cell activation, and ameliorated visual deficits.^7^^,^^10^ In this study, we compared the intravitreal injection of live ASCs (using the best tolerated dose of 1000 cells/eye) and the intravitreal injection of ASC secretome for their benefits using the same mTBI model, testing benefit for up to 10 months after blast injury (Fig. 2A). Intravitreal injection of ASCs and ASC-CCM was well tolerated in all groups of mice. All animals survived the single blast injury for 10 months. None of the blast injury mice either receiving saline solution or ASC-CCM developed any visible abnormalities as confirmed by fundus imaging and OCT (Supplementary Fig. S2). One animal out of nine in the sham-blast injury group had vitreous haze because of the injection and therefore was excluded from the study. Three animals of eight in the blast injury with ASC group showed vitreous haze or deposition of cells in the vitreous; but the underlying retina was intact with no retinal detachment or neovascularization at 10 months after intravitreal injection, and they were therefore included in the study (Supplementary Fig. S2).

Visual function in all groups of mice was assessed by Optomotry at five and 10 months after blast injury. Previously we have shown that in the focal cranial blast model of mTBI, mice show bilateral visual deficits at one month after blast injury.^25^^,^^27^^–^^29^ In the present study, we further showed that blast mice demonstrated long-lasting visual deficits, namely at both five and 10 months after blast injury, as compared to their age-matched sham blast mice (Figs. 2, 3). Furthermore, both ASC and ASC-CCM mice showed significant improvement in both visual acuity and/or contrast sensitivity thresholds compared to blast injury mice at five or 10 months after blast injury. Either due to age-associated changes in visual performance or change in the investigator involved in the analysis, the baseline OKN readings at 10 months were slightly higher than those at the five-month period; however, these subtle changes occurred across all groups of mice and therefore were determined to have little to no effect on the outcome reported here.

Sham mice had a visual acuity of 0.412 ± 0.0 c/d in the left eye and 0.406 ± 0.0 in the right eye (Fig. 2B) at 5 months and after 10 months visual acuity was 0.480 ± 0.01 c/d in the left eye and 0.483 ± 0.0 in the right eye (Fig. 2C). On the other hand, the visual acuity in blast mice receiving saline at five months after blast was significantly decreased when compared with age-matched sham mice in both left eyes (0.320 ± 0.0 vs. Sham: 0.412 ± 0.0 c/d, *P* < 0.001) and right eye (0.348 ± 0.0 vs. Sham: 0.406 ± 0.0 c/d, p < 0.001), (Fig. 2B). Visual acuity in blast mice receiving saline solution at 10 months after blast injury was also significantly decreased when compared with age-matched sham mice in both left eye (0.398 ± 0.0 vs. Sham: 0.480 ± 0.01 c/d; *P* < 0.001) and right eye (0.373 ± 0.01 vs. Sham: 0.483 ± 0.0 c/d, *P* < 0.001 (Fig. 2C). Interestingly, mice that received ASC-CCM demonstrated a significant improvement in visual acuity compared to blast mice receiving saline at five months in the left eye (five month: 0.385 ± 0.0 vs. blast-saline: 0.320 ± 0.0 c/d, *P* < 0.001) with a trend toward significance at 10 months after blast injury that is not significantly different than in Sham mice (10 months: 0.450 ± 0.02 vs. blast-saline: 0.398 ± 0.0 c/d, *P* = 0.06; vs. sham-saline: 0.480 ± 0.01 c/d, *P* > 0.05). In the case of right eyes, mice that received ASC-CCM demonstrated marginal improvement in visual acuity compared to blast mice receiving saline that did not reach significance in the right eye at five months (0.36 ± 0.01 vs. blast-saline: 0.348 ± 0.0 c/d, *P* = 0.2), but demonstrated significance at the 10-month time point (0.455 ± 0.01 vs. blast-saline: 0.373 ± 0.01 c/d, *P* < 0.001) (Figs. 2B, 2C). Unlike in the case of mice receiving ASC-CCM, mice receiving ASCs demonstrated a significant improvement in visual acuity compared to blast mice receiving saline at five months in both the left eye (0.352 ± 0.0 vs. blast-saline: 0.320 ± 0.0 c/d, *P* < 0.01) and right eye (0.382 ± 0.0 vs. blast-saline: 0.348 ± 0.0 c/d, *P* < 0.001). By contrast, no significant improvement was noted at 10 months after blast injury compared to blast-saline mice for the left eye (0.401 ± 0.02 vs. blast-saline: 0.398 ± 0.0 c/d, *P* = 0.44), but a significant improvement compared to blast-saline mice was seen for the right eye (0.450 ± 0.02 vs. blast-saline 0.373 ± 0.01, *P* < 0.01) (Figs. 2B, 2C).

Contrast sensitivity thresholds of blast mice receiving saline showed an increase in the contrast needed to detect 0.042 c/d in both eyes compared to their age-matched sham-blast injury mice receiving saline at five months (left eye: 77.8 ± 14.6 vs. Sham: 8.9% ± 0.7%, *P* < 0.001; right eye: 52.7 ± 5.2 vs. sham: 8.8% ± 0.7%, *P* < 0.001) and 10 months (left eye: 63.2 ± 7.5 vs. sham: 12.5% ± 9.2%, *P* < 0.001; right eye: 77.9 ± 11.1 vs. Sham: 11.0% ± 9.5%, *P* < 0.001) after blast injury. Similarly to the visual acuity data, mice receiving ASC-CCM demonstrated a significant improvement in contrast sensitivity when compared to blast mice receiving saline solution at five months and at 10 months after blast in the left eye (five months: 34.8 ± 2.5 vs. blast-saline: 77.8% ± 14.6%, *P* < 0.001; 10 months: 32.8 ± 8.3 vs. blast-saline: 63.2% ± 7.5%, *P* < 0.010) with no change in the right eye at five months (50.4 ± 11.6 vs. blast-saline: 52.7% ± 5.2%, *P* = 0.400) but with a significant improvement at 10 months (25.0 ± 5.0 vs. blast-saline: 77.9% ± 11.1%, *P* < 0.001) (Figs. 2D, 2E). Finally, mice receiving ASC demonstrated a significant improvement in left eye contrast sensitivity when compared to blast mice receiving saline at five months (56.4 ± 4.6 vs. blast-saline: 77.8% ± 14.6%, *P* < 0.01) and trended toward significant improvement at 10 months after blast injury (37.8 ± 14.0 vs. blast-saline: 63.2% ± 7.5%, *P* = 0.070). For right eyes, the contrast sensitivity threshold demonstrated a trend toward significant improvement at the five-month time point (42.8 ± 7.5 vs. blast-saline: 52.7% ± 5.2%, *P* = 0.100) and a significant improvement at the 10-month time point (39.4 ± 14.8 vs. blast-saline: 77.9% ± 11.1%, *P* < 0.050) (Figs. 2D, 2E).

Previously we have shown that ASC-CCM suppresses visual deficits after blast injury at one month.^10^ In the present study, we assessed the long-term effects of blast injury on dark-adapted scotopic ERG responses in the left eyes of blast mice with and without the intravitreal injections of ASCs and ASC-CCM at five- and 10-month post-blast time points (Figs. 3A–D). With increasing light intensities, an expected increase in b-wave amplitudes could be discerned in all groups, but with a reduction in the blast-saline mice as compared to sham blast mice across all light intensities (0.01–1.0 cd·s·m^2^). For example, the b-wave amplitude at the 1.0 cd.s.m^2^ light intensity in the blast-saline mice demonstrated a trend toward a decrease compared to sham-blast injury mice at the five-month time point (404.71 ± 17.37 µV vs. Sham: 421.95 ± 18.51 µV, *P* > 0.05), and at the 10-month time point, the difference in b-wave amplitude at the 1.0 cd · s · m^2^ light intensity between blast-saline and sham-saline mice (275.04 ± 25.75 µV vs. Sham: 338.46 ± 24.18 µV, *P* < 0.05) reached statistical significance, suggesting long-lasting effects of a single blast in the mTBI model. The a-wave amplitudes in blast mice receiving saline showed a similar increasing deficit with time, with no significant reduction at the 1.0 cd · s · m^2^ light intensity at five months compared to sham blast mice (−218.13 ± 9.79 µV vs. Sham: −228.36 ± 9.78 µV, *P* = 0.234), but with a significant difference between blast-saline and sham-saline mice at the 10-month post-blast time point (−151.49 ± 3.71 µV vs. Sham: −190.81 ± 10.95 µV, *P* < 0.05) .

At five months after blast injury, intravitreal injection of ASCs and ASC-CCM resulted in improvement in the b-wave amplitudes across all intensities. Although the b-wave amplitude at the 1.0 cd · s · m^2^ light intensity in the left eye of blast mice receiving ASC-CCM demonstrated significant improvement, the left eyes of those mice receiving ASCs failed to reach statistical significance compared to blast mice receiving saline injections (ASCs 414.01 ± 11.3 vs. blast-saline 404.71 ± 17.37 µV at 1.0 cd · s · m^2^, *P* > 0.05; ASC-CCM 430.01 ± 9.16 vs. blast-saline 404.71 ± 17.37 µV at 1.0 cd · s · m^2^, *P* < 0.05). Additionally, blast mice that received intravitreal injection of ASC-CCM, but not blast mice receiving ASCs, were insignificantly (*P* > 0.05) different from sham-blast mice suggesting the superiority of ASC-CCM. A similar effect was noted with a-wave amplitudes at five months after blast injury, with the left eyes in blast mice receiving ASC-CCM significantly different from those in blast injury mice, but not blast-ASC mice (ASC-CCM: −243.26 ± 4.47 vs. blast-saline: −218.13 ± 9.79 µV at 1.0 cd · s · m^2^, *P* < 0.05; ASCs −231.93 ± 6.89 vs. blast-saline: −218.13 ± 9.79 µV at 1.0 cd · s · m^2^, *P* > 0.05).

At 10 months after blast injury, intravitreal injection of ASC-CCM but not ASCs resulted in a modest left eye improvement compared to blast mice receiving saline in the b-wave amplitudes across all intensities despite none of the values reached significance (ASCs 268.3 ± 21.67 vs. blast-saline: 262.22 ± 23.34 µV at 1.0 cd · s · m^2^, *P* > 0.05; ASC-CCM 288.66 ± 11.76 vs. blast-saline: 275.04 ± 25.75 µV at 1.0 cd · s · m^2^, *P* > 0.05). A similar effect was noted with a-wave amplitudes at 10 months after blast injury with mice receiving ASCs and those receiving ASC-CCM both not different from blast mice receiving saline (ASCs −162.03 ± 18.04 vs. blast-saline: −151.49 ± 3.71 µV at 1.0 cd · s · m^2^, *P* = 0.32; ASC-CCM −155.17 ± 10.06 vs. blast-saline: −151.49 ± 3.71 µV at 1.0 cd · s · m^2^, *P* = 0.42).

### Glial Hypertrophy After Intravitreal Injection of ASC-CCM and ASCs in Mice Subjected to Blast Injury

**Figure 4.**
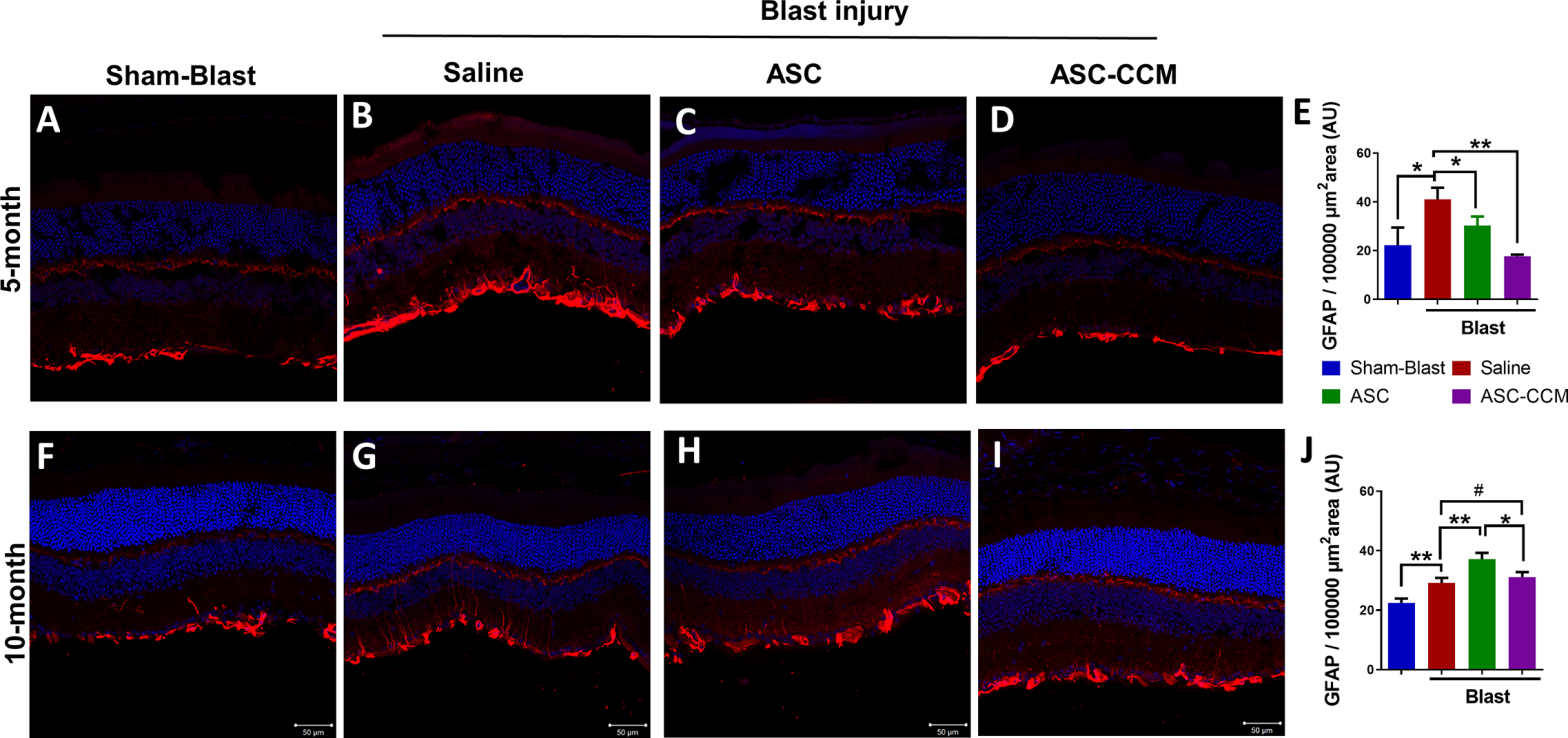
ASC-CCM and ASCs in mice subjected to blast injury partially suppress glial hypertrophy. Confocal microscope images of retinal tissue immunolabeled for GFAP in **(A)** sham mice receiving saline solution, five months; **(B)** mTBI mice receiving saline solution, five months; **(C)** mTBI mice receiving ASCs, five months; **(D)** mTBI mice receiving ASC-CCM, five months. **(E)** Image J quantification of GFAP intensity in immunolabeled retinas at five months. **(F)** Sham mice receiving saline solution, 10 months. **(G)** mTBI mice receiving saline solution, 10 months. **(H)** mTBI mice receiving ASCs, 10 months. **(I)** mTBI mice receiving ASC-CCM, 10 months. **(J)** Image J quantification of GFAP intensity in immunolabeled retinas at 10 months. *Scale bars* for A-I: 50 µm. Data represent mean ± SEM from n = 5-8 animals/group. *P < 0.05; **P < 0.01; ^#^*P* > 0.05.

Müller cell reactive gliosis is closely associated with increased GFAP immunostaining in hypertrophied Müller cells, which itself is directly linked to deleterious effects on tissue function and regeneration of retinal tissue.^30^ As in our previous studies showing increased retinal GFAP at one month after blast injury,^7^^,^^27^ in the present study we noted upregulation of GFAP expression in Müller glia in the blast injured mice that received saline both at five (Figs. 4A–E) and 10 months after blast (Figs. 4F–J). In the sham blast group (Figs. 4A, 4F), GFAP expression was only observed in Müller cells in the nerve fiber layer, NFL, whereas in the blast group that received saline, a markedly upregulated GFAP immunolabeling of Müller cell processes extending into the inner retina was observed (Figs. 4B, 4G). The presence of Müller glia processes extending into the inner retina as late as 10 months after blast injury suggests that the single focal cranial blast causes sustained retinal injury in the mice.

Intravitreal treatment with live ASCs or ASC-CCM in mice subjected to blast injury attenuated glial activation at five months after blast injury (Figs. 4C, 4D). The mean total pixel intensity of GFAP expression in the normal sham group retina was 22.3 ± 7.3 µm^2^, while the mean total pixel intensity of GFAP expression for the blast group with saline was 41.0 ± 4.8 µm^2^ (*P* < 0.05). By contrast, blast mice receiving ASC-CCM (17.8 ± 0.6 µm^2^; *P* < 0.01), as well as those receiving the ASCs (30.3 ± 3.7 µm^2^; *P* = 0.05), showed reduced GFAP expression compared to blast mice receiving saline solution (Fig. 4E).

At 10 months after blast injury, in contrast to the results at the 5-month time point, the blast mice that received ASC-CCM failed to show reduced GFAP expression compared to blast-saline mice (Fig. 4I), suggesting that ASC-CCM effects on GFAP immunolabeling were transient. Similarly, blast injury mice that received ASCs also failed to show reduced GFAP levels, and instead showed GFAP expression (Fig. 4H) that was significantly greater than in any other group. For example, the mean total pixel intensity of GFAP expression in the normal sham mice retina was 22.5 ± 1.6 µm^2^, whereas for the blast group with saline solution it was 29.2 ± 2.1 µm^2^ (*P* < 0.01). The blast mice with ASC-CCM did not show any reduction in GFAP expression compared to blast-saline mice (31.1 ± 2.1 µm^2^; *P* > 0.05). The blast mice injected intravitreally with ASCs, however, showed significantly increased GFAP levels compared to all three other groups at the 10-month time point (37.2 ± 2.6 µm^2^; *P* < 0.01) (Fig. 4J).

### Pro-inflammatory Gene Expression in Mice Subjected to Blast Injury After Intravitreal Injection of ASC-CCM and ASCs

**Figure 5.**
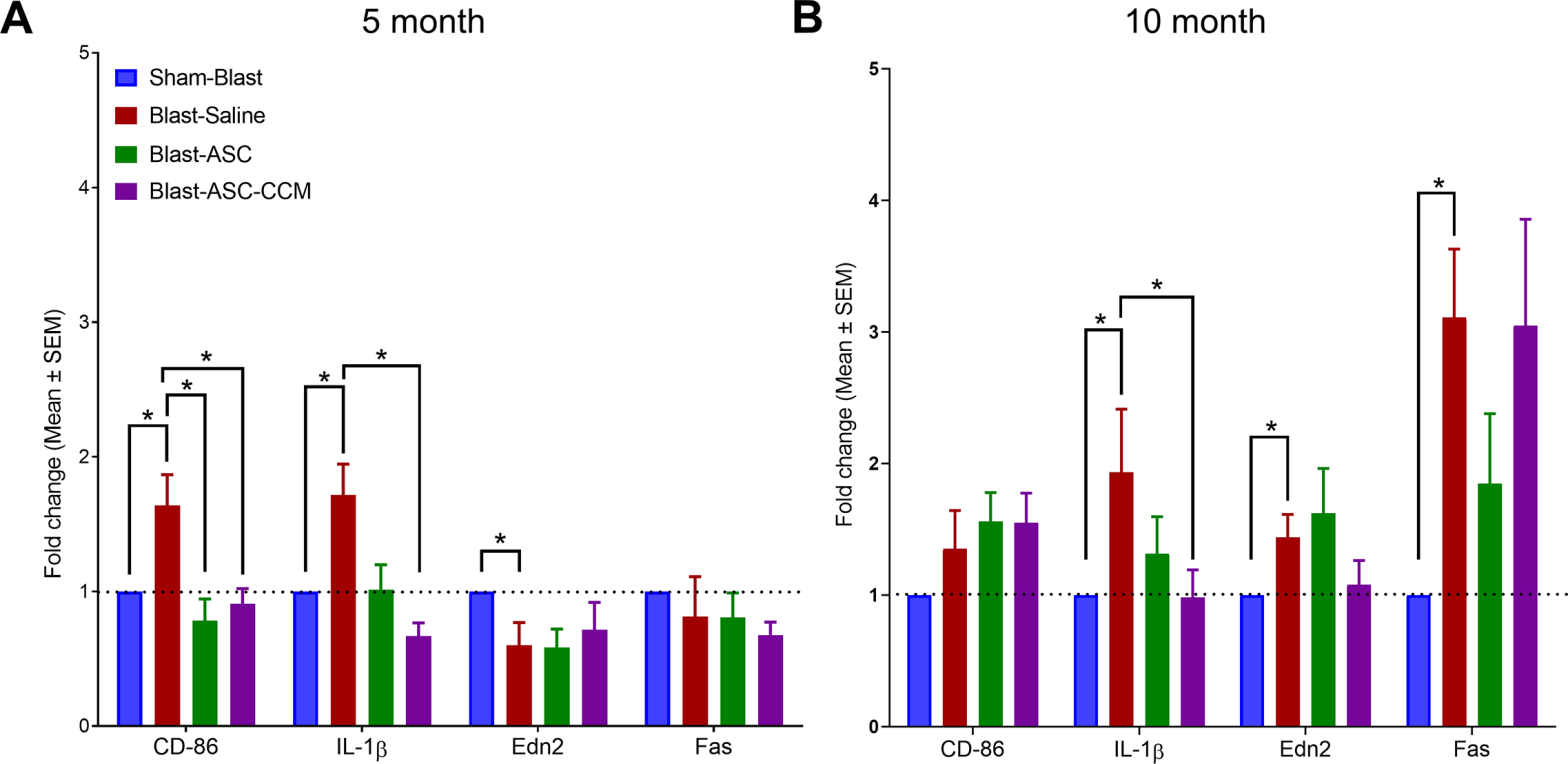
ASC-CCM and ASCs modulate pro-inflammatory gene expression in mice subjected to blast injury. Assessment of gene expression by TaqMan qPCR at five months **(A)** and 10 months after blast injury **(B)**. Changes in target gene transcripts expressed as fold change normalized to sham-blast mice. Data represent n = 6–10 animals/group. Only significant values are shown: *P < 0.05.

Changes in the expression of pro-inflammatory gene in the retina shortly after blast injury indicate the occurrence of microglial polarization and neuroinflammation, as described by us previously.^7^^,^^10^^,^^28^ Because of the potential role of these processes in the pathogenesis of post-traumatic injury, we have assessed pro-inflammatory gene changes at five and 10 months after blast injury to evaluate the long-term anti-inflammatory effects of ASC-CCM or live ASCs by assessing the expression of early and late markers of the M1 phenotype of microglia and other neuroinflammatory markers by real-time qPCR (Fig. 5).

At five months after blast injury, retinal extracts from blast mice receiving saline had increased expression of gene transcripts associated with microglial activation (Cluster of differentiation 86, CD-86: 1.64 ± 0.18, *P* < 0.01; interleukin 1-beta, IL-1β: 1.71 ± 0.14, *P* < 0.05) compared to sham-blast mice (normalized to 1.0 ± 0 for all gene transcripts; Fig. 5A). In contrast, retinal extracts from mice receiving ASC-CCM, as well as those receiving ASCs demonstrated a significant reduction in CD-86 (ASC: 0.78 ± 0.16, *P* < 0.01; ASC-CCM: 0.90 ± 0.11, *P* < 0.01) and IL-1β (ASC: 1.01 ± 0.18, *P* < 0.05; ASC-CCM: 0.66 ± 0.08, *P* < 0.01) gene expression compared to blast mice receiving saline. At 10 months after blast injury, retinal extracts from blast mice receiving saline solution had increased expression of gene transcripts indicative of microglial activation (IL-1β: 1.93 ± 0.48, *P* < 0.05; CD-86: 1.35 ± 0.29, *P* = 0.1) and neuroinflammation (TNF receptor superfamily member 6, Fas: 3.10 ± 0.46, *P* < 0.01; Endothelin-2, Edn2: 1.43 ± 0.16, *P* < 0.05) compared to sham-blast mice (Fig. 5B). This finding demonstrates that microglial activation and neuroinflammation are long-lasting effects after a single focal cranial blast injury, although the gene expression changes at the 10-month time point were not as robust as those observed at the five-month time point. In contrast to the five-month data, retinal extracts from mice receiving ASC-CCM only showed reduced IL-1β (0.98 ± 0.22, *P* < 0.05) and Edn2 (1.07 ± 0.18, *P* = 0.09), with no change in CD-86 (1.54 ± 0.22, *P* > 0.05) or Fas expression (3.04 ± 0.80, *P* > 0.05), compared to blast mice that received saline solution. On the other hand, retinal extracts from mice receiving ASCs showed no effect on IL-1β (1.31 ± 0.28, *P* > 0.05), CD-86 (1.56 ± 0.21, *P* > 0.05), Fas (1.85 ± 0.46, *P* > 0.05) or Edn2 (1.62 ± 0.29, *P* > 0.05) compared to blast mice that received saline, suggesting a poor outcome in terms of reducing retinal inflammation with live cells.

### Safety and Toxicity in the Eye and Peripheral Organs After Intravitreal Injection of ASCs and ASC-CCM

**Figure 6.**
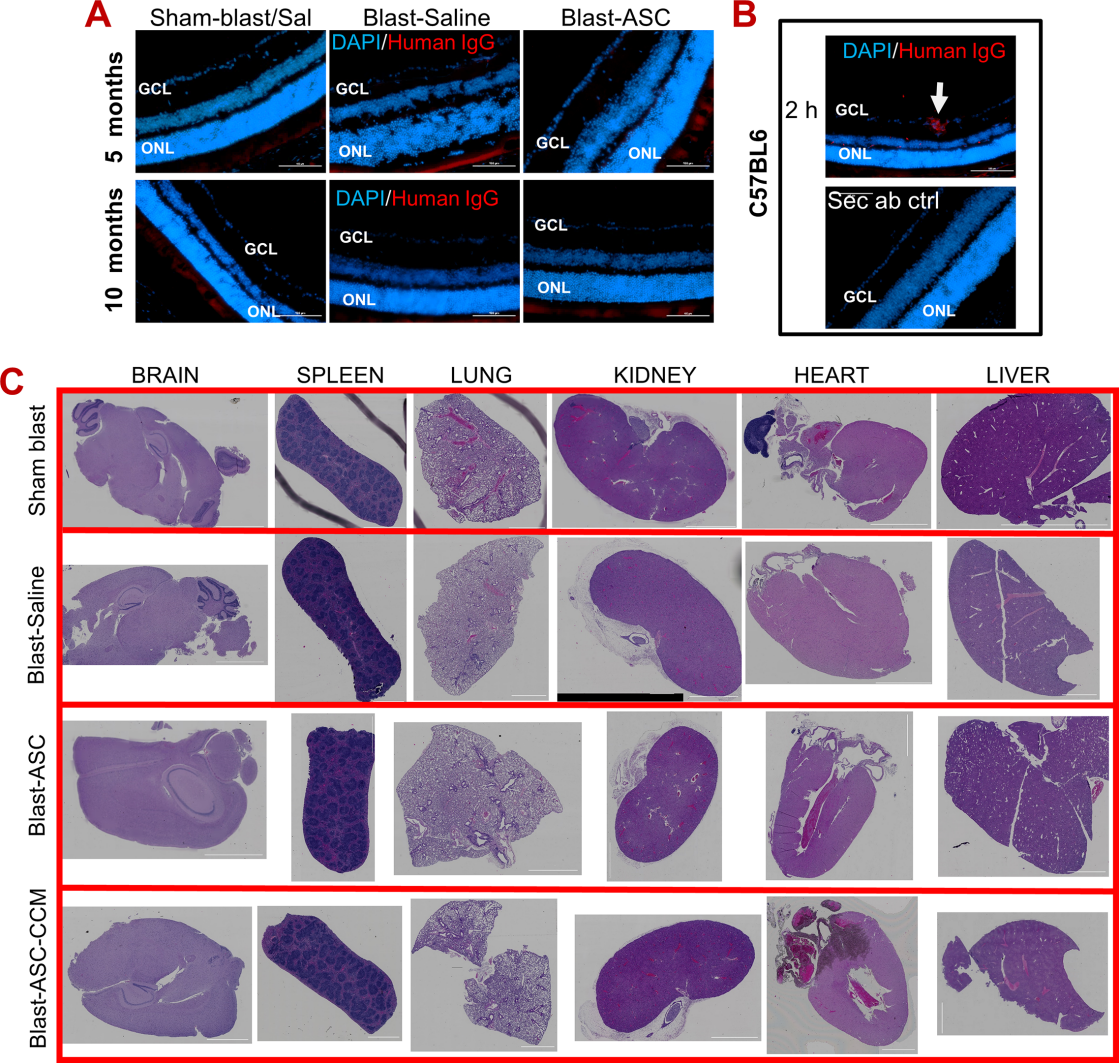
Intravitreal injection of ASCs and ASC-CCM are safe and do not cause toxicity in the eye and peripheral organs. **(A)** Representative immunofluorescence images of retina from mTBI mice treated with saline or ASCs at five and 10 months after intravitreal injection. Human ASCs were attempted to track within the retina using anti-human IgG antibodies. **(B)** Representative immunofluorescence images of retina from C57BL/6 mice treated with 20,000 ASCs at two hours after intravitreal injection. Human ASCs were detected in the GCL (*white arrow*). *Scale bars* for A, B: 100 µm. Data represent n = 5 animals/group. **(C)** Representative H&E sections of peripheral organs from all groups of mTBI mice. Data represent n = 5–6 animals/group except sham-blast (n = 3).

ASCs injected intravitreally have been shown to integrate into retina in some studies,^31^^,^^32^ whereas other studies have not observed them to integrate into the retina.^11^^,^^13^ Here we assessed if human ASCs at a dose of 1000 cell per eye injected into the mouse retina after blast injury are trackable after five and 10 months after blast injury. Histological evaluation to detect ASCs in these retinas using anti-human IgG failed to identify any human cells (Fig. 6A). As a positive control, C57BL/6 mice injected with 20,000 ASCs per eye (∼20 times more than used in our experimental mice used in the TBI treatment studies) and euthanized two hours after the intravitreal injection were examined. In these, we successfully detected human ASCs in retina, as evidenced by immunolabeling with an anti-human IgG antibody (Fig. 6B).

Several studies have shown the safety profile of MSCs delivered systemically, either in preclinical studies or clinical trials.^33^ Here, we assessed if the intravitreal injection of ASCs or their secretome cause any adverse outcomes in peripheral organs. To this end, major internal organs, including brain, spleen, lung, kidney, heart and liver, were examined for any evidence for infarction, hemorrhage, or pathologic lesion, such as tumor formation, after 10 months after intravitreal injection in the blast injury mice and compared them to blast injury mice receiving saline and sham-blast mice. H&E-stained tissue sections of the entire organs assessed for histopathological abnormalities did not show any significant abnormality related to ASCs or their secretome injections. No evidence of abnormal cells and or tissue that do not belong to the organs was noted (Fig. 6C). One of six animals in blast-ASC group showed liver steatosis and one animal developed focal, hepatic, necrotizing inflammation unrelated to stem cell injection. Focal, remote, subcapsular ischemic necrosis of hepatocytes was also noted in the Sham-blast group, suggesting age-associated changes. Similarly, one of six animals in the blast-ASC-CCM group and blast-saline groups showed focal, subpleural, histiocytic/mesothelial proliferation unrelated to stem cell therapies.

## Discussion

To the best of our knowledge, this study represents the first to report the long-term safety and efficacy of intravitreal delivery of ASCs or their secretome on visual deficits after blast injury using a focal cranial blast model of TBI. Our study reports that human ASCs at 1000 cells/eye are safe and effective in the mouse eye, with a higher number (>10,000) of cells causing retinal damage. Although our previous studies have shown visual deficits at one-month after blast injury,^7^^,^^10^^,^^24^^,^^25^^,^^27^^,^^28^^,^^34^ our current study shows visual deficits persisting in the model at five and 10 months after blast injury. Interestingly, blast mice receiving ASCs or ASC-CCM showed significant improvement in visual function at five and 10 months after blast injury and injection in the left eye (blast delivery side). However, glial hypertrophy and proinflammatory gene changes in the retina were not suppressed in blast mice receiving ASCs at 10 months after blast injury. Although human ASCs injected at 1000 cells/eye were not detectible in the retina at five and 10 months after injection, intravitreal injections of ASCs or ASC-CCM were found to be safe, with major internal organs, including the eyes, demonstrating no gross histopathological changes. The minor necrotizing inflammation, ischemic necrosis, or focal, subpleural, histiocytic/mesothelial proliferation were noted in mice from all groups and therefore determined to be unrelated to stem cell injection.

Stem cell transplantation holds great promise for retinal diseases; however, the complexity of retinal cell types and the requirement for a clear vitreous domain for visual perception in the retina provides a challenging environment for successful intravitreal stem cell therapies. Intravitreal injections have become the mainstay for treating many ocular conditions, with the delivery of biologics such as anti-VEGF, for example, achieving tremendous success.^35^ In line with other ocular cell therapies, we and others have administered ASCs or their secretome via intravitreal injections to deliver them close to the damaged retinal vasculature and neurons.^4^^,^^7^^,^^11^^,^^13^^,^^31^ Although the majority of MSC therapeutics in nonocular models are delivered systemically via intravenous delivery,^36^ it should be noted that a study of MSC intravenous infusion into a rodent model of TBI did not find it beneficial,^37^ highlighting the potential shortcomings of intravenous therapy. Because intravitreal delivery represents local delivery, confined by the blood-retina barrier, it avoids the first-pass effect of lung entrapment.^37^ Additionally, local delivery requires a low dose of stem cell therapeutics, as observed in our studies. Our data show the feasibility of delivering live ASCs for intravitreous injection in a safe and effective manner; however, caution must be exercised using the live cells considering their potential shortcomings reported here. On the other hand, we show that ASC concentrated secretome is safe and effective in the TBI model for up to 10 months after intravitreal injection. Although we have not tested different doses of ASC-CCM in this study, based on this and our previous study using a direct ocular blast injury model,^12^ it is safe to assume that 1 to 2 µL of ASC-CCM (∼200 ng total protein) in a mouse eye is safe and effective in TBI models. Because our studies are conducted with ASCs derived from a single donor, future studies should determine the effects of repeated dose (to improve efficacy) and donor variability, in addition to delayed intervention, to better translate these stem cell therapeutics into the clinic.

Although our results point to ASC-CCM being well-tolerated, safe, and effective for preventing vision loss due to mTBI, ASCs need to be carefully assessed for a safe and effective outcome. While the integration of stem cells into the host tissue suggests that they are functional, intravitreal injection of ASCs in rodent models has either shown them to integrate^31^^,^^32^ or not integrate into the retina.^11^^,^^13^ Histological evaluation to track ASCs in the retina in our studies using either human IgG (Fig. 6) or human nuclear antigen antibody (data not shown) failed to detect ASCs injected into the vitreous cavity at the end of five or 10 months after blast injury, but also when examined one month after blast injury (Supplementary Fig. S3). Because we only injected 1000 cells per eye, it is likely that there is an insufficient number of cells to be detected by our technique, or cells did not integrate or washed away during retinal preparation for histology. The integration of cells is a rare event, and most cells, if any, are either stuck to the lens or possibly taken up by immune cells by a process known as efforocytosis.^38^ It is worthy to note that intravitreal injection of 20,000 cells per eye (×20 of the experiment) demonstrated very few cells that had integrated into the retina, suggesting that the ASCs most likely function via their trophic factors. Based on OCT and histological evaluation, it seems that the 1000 cells per eye are safe and effective in the long term.

In our studies comparing the efficacy of live ASCs versus ASC-CCM, we saw both similarities and differences. Both ASCs and ASC-CCM treatment in blast injury significantly alleviated the decrease in visual acuity and increase in contrast sensitivity thresholds after blast injury at five and 10 months, specifically in the left eye being the blast delivery side. Although the underlying mechanism(s) is/are not known concerning the right eye deficits in our model,^28^^,^^29^ recently it has been shown that the type of animal holder used and the compressive forces generated in blast studies affecting visual and cognitive outcomes^28^^,^^39^^,^^40^ might be involved in our studies. Consequently, left and right eye injuries may have different time course and severities, and different treatment outcomes due to the different bases. Interestingly, mice receiving ASC-CCM performed better than those mice receiving ASCs in vision function tests in both the eyes, including OKN and ERG studies. Müller gliosis is known to be a hallmark of eye disease or injury, including that stemming from mTBI, and such hypertrophied Müller cell processes are known to impede the therapeutic benefit of several approaches ranging from ocular gene therapy to cell therapies to electronic implants.^41^^–^^43^ Extending on our previous observation at one month after blast injury, ASC-CCM was found to be effective in reducing GFAP processes at the five-month post-blast injury time point but had no effect at 10 months. On the other hand, mice receiving live ASCs showed reduced GFAP immunoreactivity at five months but a surprising increase in GFAP immunoreactivity at 10 months after blast injury. It is unclear if this increase in GFAP expression in mice receiving ASCs is a sign of regeneration, as previously reported in the brain tissue of a repetitive blast model,^44^ or because of changes in JAK/STAT3 cascade,^43^ resulting in increased reactive gliosis and neuroinflammation. In this regard, our evaluation of mRNA expression of genes associated with glial activation and neuroinflammation in the retinal tissue demonstrated better outcomes with ASC-CCM compared to those receiving ASCs. For example, IL-1β, a pro-inflammatory cytokine that promotes microglial activation was shown to be increased in blast injury even after five and 10 months compared to their age-matched sham blast group. Such an increase in IL-1β was noted in brain tissue as early as one hour after injury,^45^ with sustained levels reported even after three months after TBI.^46^ While blast mice receiving ASCs showed reduced IL-1β expression only at five months, the ASC-CCM treated group showed a significant reduction in IL-1β levels at both five months and 10 months. The display of CD86 on microglia determines the activation state of microglia, with its upregulation linked to M1 microglia in several models of TBI as early as one day after injury, with sustained levels reported up to three months. Accordingly, CD86 was shown to be upregulated in mTBI at five and 10 months after blast injury. Although at five months after blast injury, both ASCs and ASC-CCM treatment resulted in a reduction in CD86 expression, at 10 months, only ASC-CCM treated mice showed the reduction, suggesting a poor outcome with live cells. A number of genes that are involved in neuroinflammation, neurotransmission, metabolism, neuroplasticity, development and aging, and neuron-glia interactions including interferon regulatory factor 8, vascular cell adhesion molecule-1 (Vcam1), erb-b2 receptor tyrosine kinase 3 (Erbb3), and glutamine synthetase that we reported earlier^7^^,^^24^ in mTBI model were shown to be differentially expressed between sham blast and blast injury, however, none of them reached statistical significance (data not shown), suggesting that molecular signaling pathways in our single blast injury model during the long-term are temporally regulated. In support of this, previously, we have shown more robust gene transcript changes at three to five days after blast injury.^7^^,^^28^

Although our studies have shown similarities and differences between live ASC or ASC-CCM treatments, our comparisons have limitations. For instance, (1) ASC-CCM injected into the mouse eye corresponds to ∼200 ng of total protein that is likely obtained from ∼10,000–15,000 ASCs in culture, although we only injected 1000 live cells into the eye. (2) The pharmacokinetic and pharmacodynamics of protein biologics such as ASC secretome are likely different from those of live cells. (3) The mechanisms of action of ASCs and ASC-CCM likely differ from each other. Although live ASCs require preconditioning to secrete bioactive molecules in response to the local blast injury environment, ASC-CCM preconditioned with cytokines in a culture likely has the bioactive molecules readily available. (4) The live ASC strategy has limitations because of the viability and secretory capacity of stem cells. On the other hand, secretome therapy requires repeated dosage to have a prolonged effect. (5) We note few incompatibilities in ERG and optokinetic measurements; for example, blast mice receiving ASC at the five-month time point demonstrated significant improvement in contrast sensitivity thresholds but failed to show any improvement in ERG. Similarly, blast mice receiving ASC-CCM at the 10 month time point failed to show improvement in ERG but showed improved contrast sensitivity thresholds. One potential explanation of this incompatibility may be because cell signaling underlying the retinal function (ERG) is likely different from that underlying the visual deficits (OKN) occurring along the visual-motor chain and thus may yield different outcomes. More studies are warranted, including assessing pattern ERG and visual evoked potentials and histological assessment of optic nerve axons that might help reveal the potential mechanism(s) of ASC-based therapeutics.

## Conclusions

Our studies provide evidence that intravitreal injection of ASC-CCM is safe and effective up to 10 months against visual deficits in an mTBI model. Unlike ASC-CCM, a low number of live ASCs (as low as 1000 cells/eye) alleviated visual deficits of mTBI, but live ASCs failed to show suppression of glial hypertrophy and pro-inflammatory gene changes in the retina at 10 months after blast injury. Considering the modest rescue in visual function and the risk of retinal damage with live cells, our studies suggest that ASC-CCM has better efficacy and safety profile to live cells for the treatment of visual dysfunction in mTBI.

## Supplementary Material

Supplement 1
