# Supplementary material for: A Long-Term Safety and Efficacy Report on Intravitreal Delivery of Adipose Stem Cells and Secretome on Visual Deficits After Traumatic Brain Injury
Source: Transl Vis Sci Technol. 2022 Oct 3;11(10):1. doi: 10.1167/tvst.11.10.1 (PMC9547363; doi:10.1167/tvst.11.10.1)
Supplement: Supplement 1 [file tvst-11-10-1_s001.pdf]

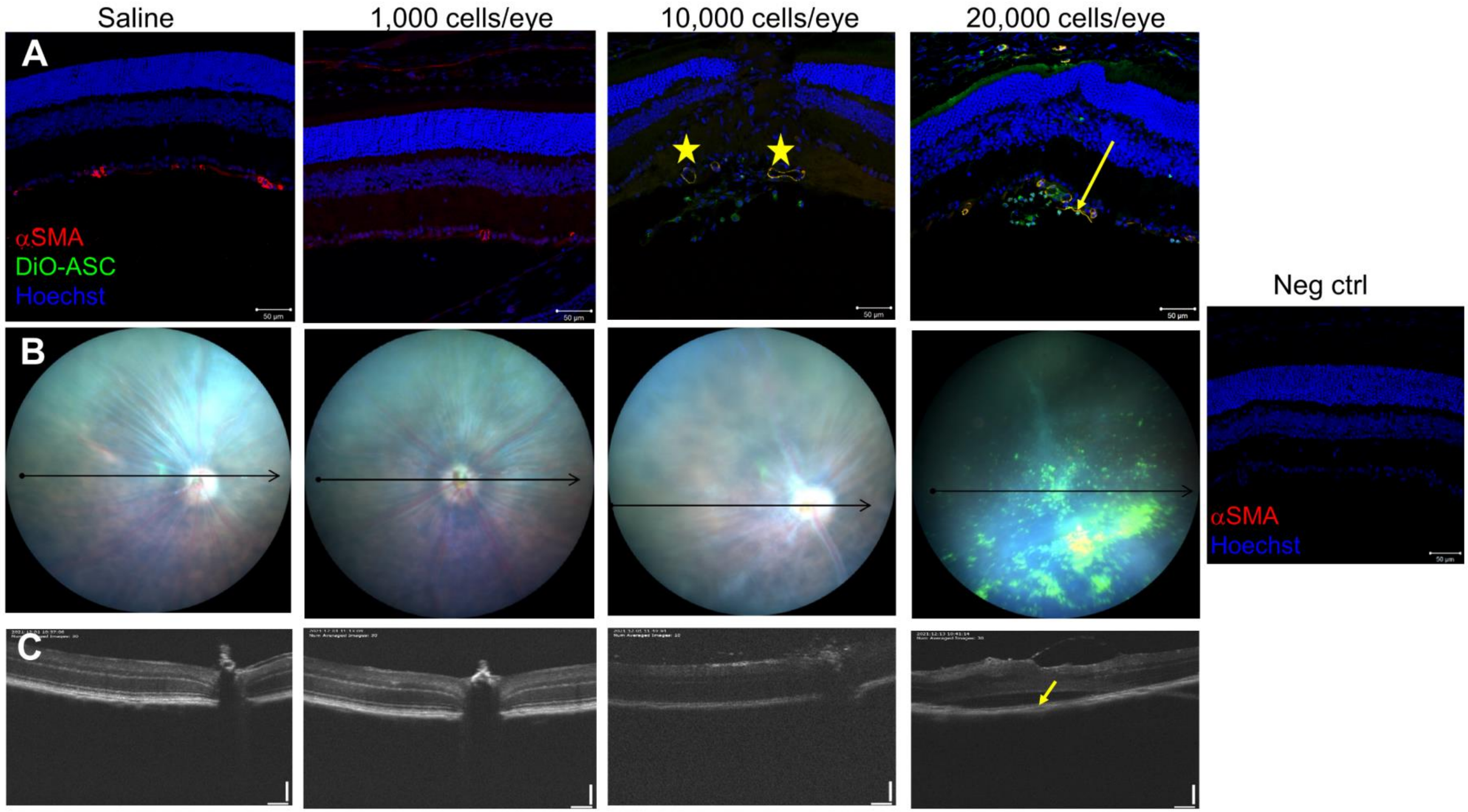

Supplemental Figure 1. **Intravitreally injected DiO-labeled ASCs are detectible in the retina and are associated with retinal damage.** (**A**). Representative confocal immunofluorescence images of C57BL/6 mice retina intravitreally injected with saline or increasing doses of DiO labeled ASCs. * Indicates DiO labeled ASC double-positive for αSMA. Arrow indicates fibrotic growth. Scale bar = 50 µm. (**B**). Representative en-face images of OCT scan showing the b-scan orientation (arrow). (**C**). Representative b-scan

images from different groups. Arrow indicates retinal detachment. A retinal section without primary antibody but with a secondary antibody control (Neg ctrl) shows the specificity of the antibody. Data represent n=5 animals/group.

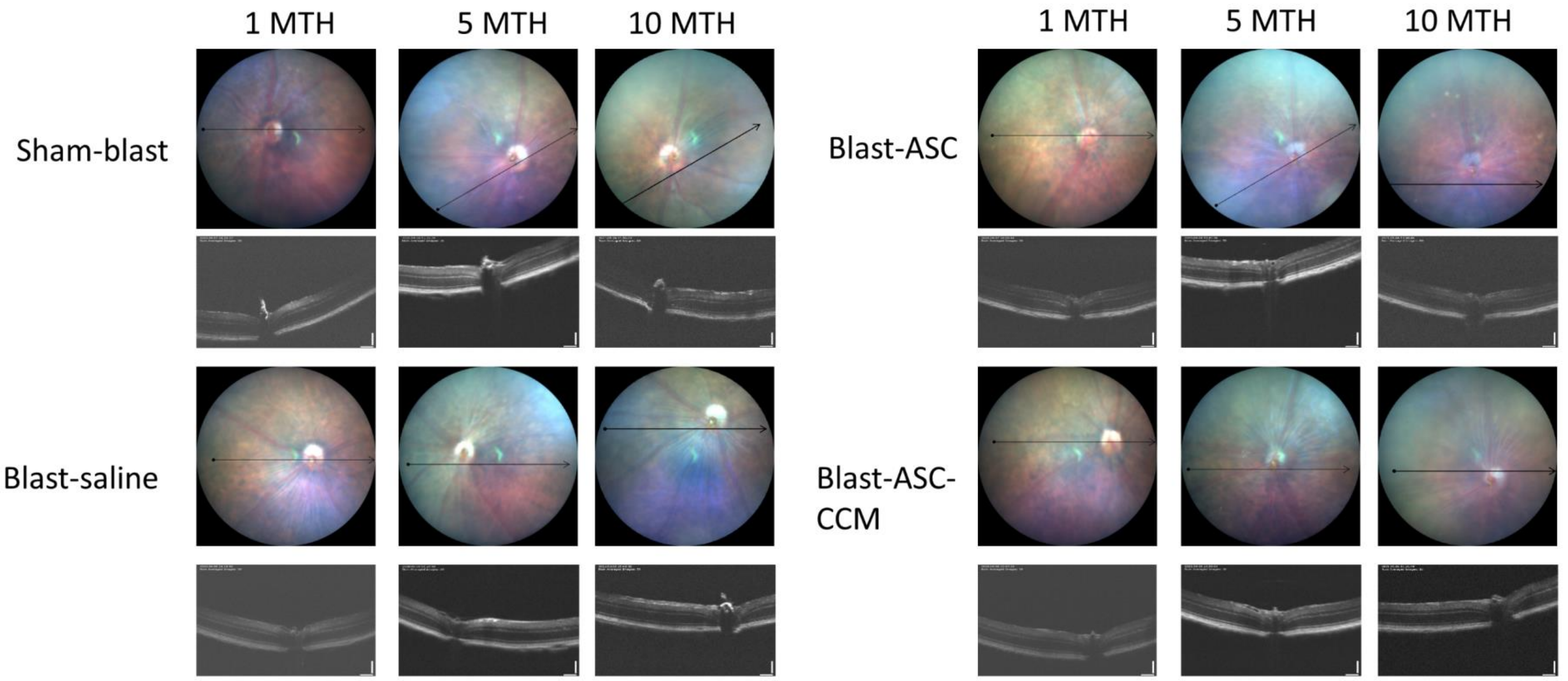

Supplemental Figure 2. Representative OCT images from mTBI mice at 1, 5, and 10-month post-blast injury. Both ASC-CCM and ASCs at 5 and 10-month post-blast injury as assessed by OCT showed no obvious safety issues. Same animals at 1, 5 and 10 months are shown for comparison. Data represents 6-8 animals/group.

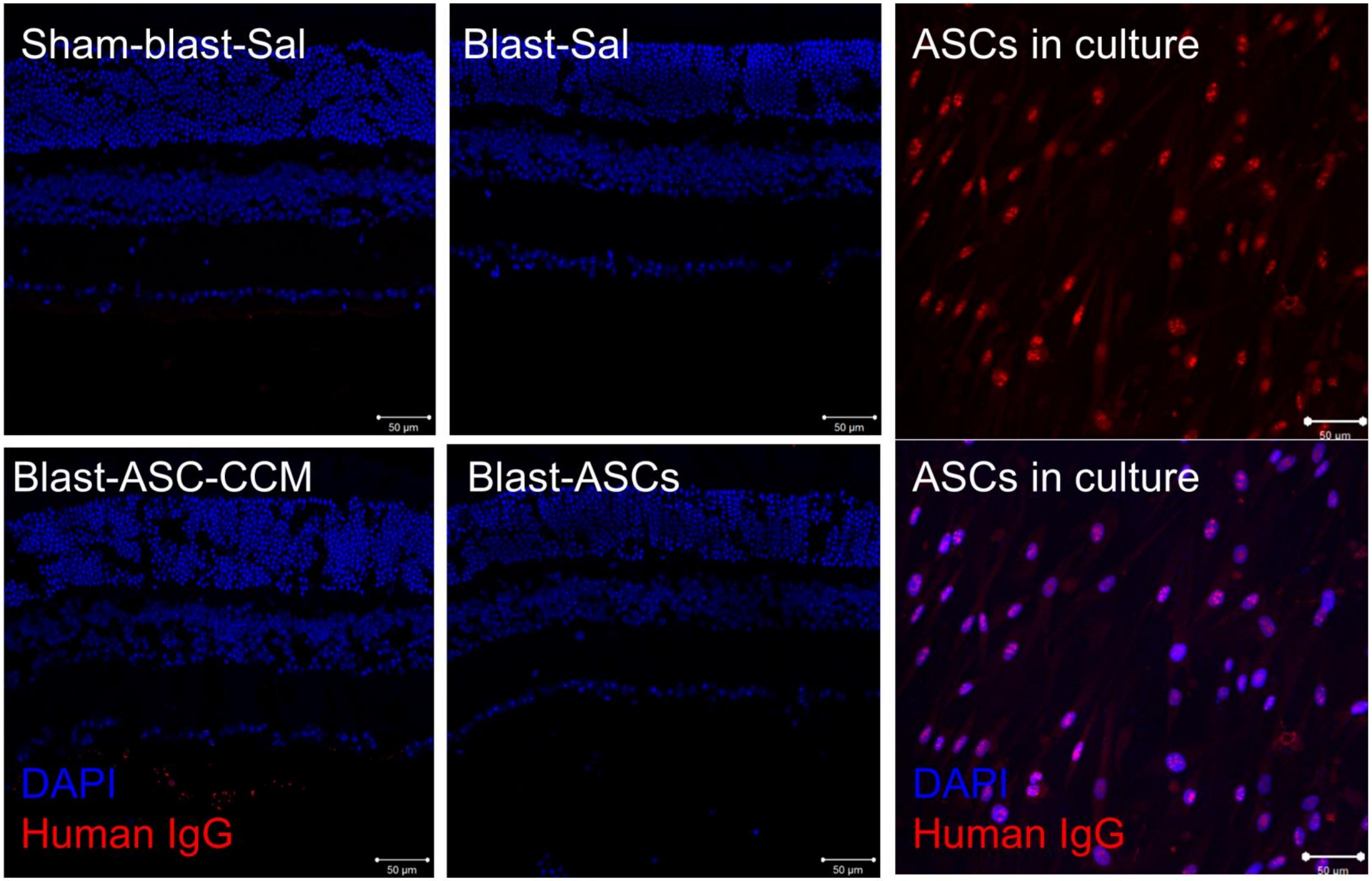

Supplemental Figure 3. ASCs failed to integrate into the blast retina at 1-month post-blast. Representative confocal images demonstrated no immunostaining for human IgG (thus no association of the ASCs with host vessels or any structures within the retina)

in all groups. Positive immunostaining is shown with human ASCs cultured *in vitro*. Data represents n=5 animals/ASCs group. ***, $p<0.001$ Scale bar=50µm.
